# Minicircle DNA Vaccines: Overcoming Delivery and Expression Barriers in Next-Generation Immunization

**DOI:** 10.3390/vaccines14070563

**Published:** 2026-06-26

**Authors:** Ibtihal S. Alduhaymi, Majed A. Majrashi, Ibrahim A. Alradwan, Faisal S. Alagrafi, Musaad A. Altammami, Ahmad M. Aldossary, Fahad A. Almughem, Abdullah A. Alshehri, Mohannad M. Fallatah, Nojoud Al Fayez, Essam A. Tawfik

**Affiliations:** 1 Advanced Diagnostics and Therapeutics Institute, Health Sector, King Abdulaziz City for Science and Technology (KACST), Riyadh 11442, Saudi Arabia; ialduhaymi@kacst.gov.sa (I.S.A.);; 2 Bioengineering Institute, Health Sector, King Abdulaziz City for Science and Technology (KACST), Riyadh 11442, Saudi Arabia; 3 Aging Research Institute, Health Sector, King Abdulaziz City for Science and Technology (KACST), Riyadh 11442, Saudi Arabia; 4 Wellness and Preventative Medicine Institute, Health Sector, King Abdulaziz City for Science and Technology (KACST), Riyadh 11442, Saudi Arabia

**Keywords:** DNA vaccines, mcDNA, infectious diseases, cancer

## Abstract

DNA vaccines have emerged as a promising immunization platform, offering key advantages over conventional vaccine approaches, including superior stability, a favorable safety profile, rapid and flexible antigen design, and scalable manufacturing. However, their clinical efficacy has remained limited, primarily due to inefficient cellular uptake, poor endosomal escape, and degradation of the plasmid DNA within host cells. Recent advances have highlighted minicircle DNA (mcDNA) as a next-generation alternative to conventional plasmid vectors. mcDNA constructs are compact, backbone-free episomal vectors containing only the expression cassette, including the promoter, transgene, and polyadenylation signal, while lacking bacterial sequences such as antibiotic resistance genes and origins of replication. This reduced vector size reduced vector-driven innate immune activation and susceptibility to epigenetic silencing, thereby improving transfection efficiency and supporting more sustained transgene expression in both dividing and non-dividing cells. This review provides a comprehensive overview of mcDNA technology in the context of vaccine development, discussing its structural design and production principles, mechanistic advantages over conventional plasmid DNA, and current applications across infectious disease and cancer vaccine platforms. In addition, we explore recent delivery strategies to enhance mcDNA transfection and immunogenicity, summarize existing limitations that hinder translation into applications, and outline future directions to optimize mcDNA-based vaccine technologies.

## 1. Introduction

DNA vaccines are an investigational immunization strategy that offers several potential advantages over conventional vaccine approaches, including high stability, rapid and flexible antigen design, cost-effectiveness, and ease of large-scale manufacturing. Despite these promising features, their clinical efficacy in humans has remained limited, largely due to low antigen expression and insufficient immune induction in vivo [1,2,3]. In addition, promoter silencing following plasmid delivery into host cells significantly compromises sustained transgene expression; even when strong viral promoters such as the cytomegalovirus (CMV) are employed, transgene expression often fails to persist long enough to achieve the desired therapeutic effect in vivo [4,5]. This silencing is mediated by epigenetic mechanisms including DNA methylation and repressive histone modifications, which promote chromatin condensation and transcriptional suppression. Promoter silencing is particularly evident in slowly dividing or non-dividing cells and may vary depending on tissue type and host immune status [6].

Another important limitation is the presence of bacterial backbone elements, including antibiotic resistance genes and immunostimulatory CpG motifs. Antibiotic resistance sequences raise biosafety concerns, particularly with respect to horizontal gene transfer and the spread of antimicrobial resistance, while CpG motifs activate Toll-like receptor 9 (TLR9) and can drive excessive innate immune responses [7,8,9,10]. Together, these limitations highlight the need for next-generation DNA vectors that retain immunogenic potency while eliminating undesirable prokaryotic elements.

To overcome these challenges, minicircle DNA (mcDNA), a non-viral vector, has emerged as a promising alternative platform for DNA vaccination and gene therapy. mcDNA is generated by removing the bacterial backbone from a parental plasmid, retaining only the eukaryotic expression cassette, including the promoter, transgene, and polyadenylation signal, while eliminating antibiotic resistance genes and bacterial origins of replication [11]. This minimized vector architecture confers a smaller molecular size, improved transfection efficiency, enhanced transgene expression, and a more favorable safety profile [12]. Consequently, minicircle vectors exhibit prolonged and sustained transgene expression in both dividing and non-dividing cells, making them particularly suitable for therapeutic applications that require durable antigen expression [11]. In addition, mcDNA circumvents key risks associated with viral vectors, such as insertional mutagenesis and vector-specific immunity [13].

Due to these unique properties, there is growing interest in mcDNA as a DNA-based vaccine platform, particularly for cancer immunotherapy and infectious disease prevention. Accumulating evidence suggests that mcDNA-based vaccines elicit stronger antigen-specific CD8^+^ T-cell responses and enhanced immunogenicity compared with conventional plasmid-based approaches [14]. These enhanced cytotoxic immune responses are especially important for effective tumor eradication. Moreover, mcDNA-based vaccines may reduce excessive TLR9 activation due to a lack of bacterial CpG motifs while still inducing sufficient innate immune responses and improved antigen cross-presentation by APCs [14,15,16]. Collectively, these features have driven intensive efforts to develop and optimize mcDNA as a next-generation vaccine platform.

This backbone-free rationale is not limited to mcDNA. In parallel, other non-plasmid DNA platforms have been developed to improve the safety, purity, and manufacturability of DNA vaccines. Doggybone DNA (dbDNA) is a bacteria-free, closed linear DNA format produced by cell-free enzymatic methods and has demonstrated cellular and humoral immune responses comparable to those of conventional plasmid DNA in preclinical vaccine models despite lacking bacterial CpG-rich backbone sequences [17,18]. Similarly, hairpin DNA (hpDNA; 4basebio) is a linear DNA platform covalently closed by single-stranded hairpin ends and manufactured using fully cell-free enzymatic methods. In preclinical neoantigen vaccine models, electroporated hpDNA elicited robust CD8^+^ and CD4^+^ T-cell responses, durable immune memory, and tumor protection, with comparable performance to plasmid DNA at lower DNA doses [19]. Together with mcDNA, these platforms reflect a broader shift toward minimized, backbone-free DNA vaccine technologies designed to reduce undesirable bacterial elements, improve manufacturing purity, and support future translational applications.

Accordingly, this review discusses the structural design and production of mcDNA; its mechanisms of action, advantages and limitations; its applications in disease prevention and treatment; and its prospects for clinical translation.

## 2. Structure and Production of mcDNA

**Figure 1:**
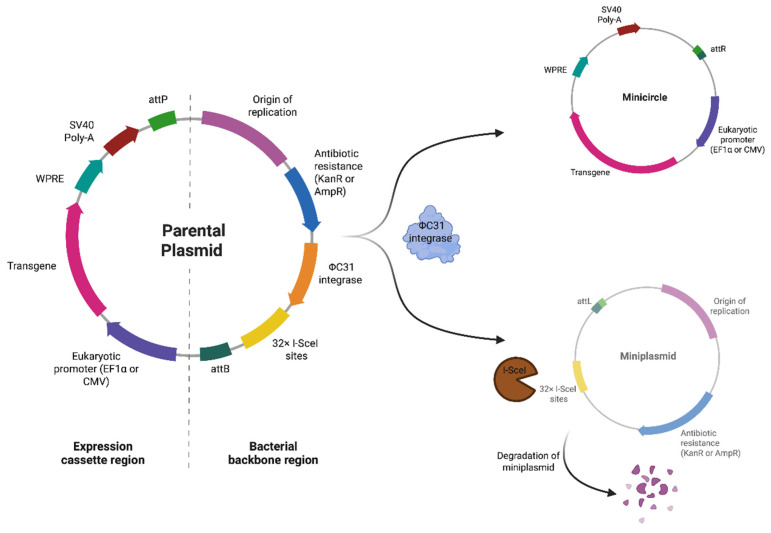
Schematic illustration of minicircle DNA production. The parental plasmid contains a eukaryotic expression cassette and a bacterial backbone. Upon induction, site-specific recombination between the attB and attP sites is mediated by the ΦC31 integrase, where the parental plasmid is split into two separate circular DNA molecules through site-specific recombination. The process generates mcDNA that retains the promoter, transgene, and regulatory elements required for expression. The remaining miniplasmid, which contains the bacterial backbone, is then cleaved by the I-SceI endonuclease and degraded, enabling purification of the mcDNA. Created in BioRender.com. Al Fayez. N (2026) https://BioRender.com/s6wxr24 (accessed on 23 June 2026).

The mcDNA is a next-generation, small, circular, supercoiled episomal vector designed to provide higher transgene expression and lower immunogenicity than conventional plasmids or viral DNA vectors [12]. As shown in Figure 1, it is generated by removing bacterial backbone elements from the parental plasmid such as antibiotic resistance genes (KanR or AmpR) and bacterial origins of replication, leaving only the eukaryotic expression cassette required for gene expression. This can be performed by site-specific recombination between the attB and attP sites which is mediated by the ΦC31 integrase. After recombination, the parental plasmid is split into two separate circular DNA molecules which are mcDNA that retains the promoter, transgene, and regulatory elements required for expression and an unwanted miniplasmid that contains the bacterial backbone which is then cleaved by the I-SceI endonuclease and degraded. The backbone-free architecture of mcDNA reduces immune recognition and supports stronger and more sustained gene expression. mcDNA remains episomal within the nucleus rather than integrating into the host genome, thereby reducing the risk of insertional mutagenesis and other integration-related safety concerns, and is increasingly investigated as a non-viral vector platform for gene therapy, DNA vaccination, and cell reprogramming applications [12].

The functional core of the mcDNA consists of a continuous expression cassette that contains a eukaryotic promoter or enhancer, the coding sequence, and a transcriptional termination and polyadenylation signal [15]; additional regulatory elements such as internal ribosome entry sites, post-transcriptional control elements, or tissue-specific promoters can be incorporated as needed [15]. Structurally, mcDNA is typically isolated as a highly supercoiled monomer, which increases compactness, facilitates interaction with cationic delivery systems, and improves cellular uptake relative to relaxed or nicked DNA. Structurally, removal of CpG-rich bacterial backbone sequences lowers the number of unmethylated CpG motifs within the vector. As a downstream biological effect, this reduced CpG burden may attenuate TLR9-mediated innate immune activation and decrease vector-driven inflammatory responses [20]. Together with the reduced vector size and supercoiled topology, these features support more efficient and durable transgene expression and contribute to a favorable safety profile, as mcDNA persists in the nucleus as a non-integrating episome rather than integrating into host chromosomes [21].

Multiple strategies have been developed for mcDNA production, ranging from bacteria-based in vivo systems to fully cell-free workflows. One widely used in vivo approach uses an engineered *E. coli* strain (e.g., ZYCY10P3S2T) that harbors a parental plasmid containing attB and attP recombination sites flanking the expression cassette and expresses a site-specific recombinase, namely the ΦC31 integrase, upon induction. Recombination at *attB* and *attP* excises the cassette as a minicircle, generating a separate bacterial backbone circle (Figure 1) [22,23]. A co-expressed restriction endonuclease (such as I-SceI) then selectively degrades the backbone circle, enriching for mcDNA [12]. Although this method can be cost-effective for large-scale production, such systems may leave residual genomic DNA, parental plasmid, or endotoxin contaminants that require rigorous downstream purification [24].

Compared with conventional bacterial production methods, bacteria-free and cell-free methods have different advantages and limitations in terms of scalability, cost-effectiveness, and purity. Bacterial systems remain attractive for large-scale production because they rely on established fermentation-based workflows and existing plasmid manufacturing infrastructure, making them relatively scalable and cost-effective [25,26]. However, bacterial production methods can cause the presence of bacterial-derived impurities such as endotoxins, parental plasmid DNA, residual genomic DNA, and bacterial backbone sequences, which require additional enzymatic digestion and chromatographic purification steps [27,28]. In contrast, bacteria-free and cell-free approaches can improve mcDNA purity by minimizing bacterial backbone contamination and reducing endotoxin content. However, bacteria-free and cell-free approaches are generally less suitable for industrial-scale manufacturing and may be limited by enzyme cost, reaction scalability, and downstream processing requirements [29].

To avoid bacterial impurities, bacteria-free and cell-free synthesis methods have been established. In one strategy, the required expression cassette is amplified by the polymerase chain reaction (PCR) and subsequently circularized in vitro to form a mcDNA molecule that contains only the functional gene without bacterial backbone sequences; residual linear DNA is then removed by enzymatic digestion, yielding highly purified mcDNA [30]. In addition, cell-free approaches such as Plasmid2MC generate mcDNA directly from plasmids without bacterial culture by using the ΦC31 integrase to excise the bacterial backbone in vitro, followed by degradation of residual linear or unwanted DNA fragments to produce high-purity, supercoiled, backbone-free mcDNA [24]. More recently, mcDNA has also been synthesized using bacteria-free methods, such as PCR with Taq DNA polymerase or rolling circle amplification (RCA) with the Φ29 DNA polymerase, in which linear DNA containing loxP sites is generated and then converted to circular mcDNA by a Cre recombinase [31].

For purification and quality control, mcDNA preparations destined for therapeutic or vaccine applications must be essentially free of bacterial impurities, including genomic DNA, residual parental plasmids, backbone circles, and endotoxins [32]. Enzymatic treatments, such as the use of T5 exonuclease, can selectively digest linear DNA while sparing circular DNA, thereby removing incomplete or unwanted products [33]. Chromatographic methods are then employed to achieve clinical-grade purity and batch-to-batch consistency. Hydrophobic interaction chromatography exploits differences in hydrophobicity and topology between supercoiled mcDNA and relaxed circular DNA and RNA contaminants, enabling efficient isolation of the supercoiled vector [32,34]. Anion exchange chromatography and HPLC further separate mcDNA from remaining impurities based on differences in charge density and size. Together, these approaches provide robust purification and quality-control frameworks suitable for the production of mcDNA for gene therapy and vaccines [35,36].

## 3. Key Advantages of mcDNA over Conventional Plasmids for DNA Vaccination

mcDNA consistently achieves superior and more sustained transgene expression in vitro and in vivo compared to the conventional plasmid DNA, a feature of particular importance for effective DNA vaccination [11,37]. Several mechanisms contribute to this effect: removal of bacterial promoters and terminators reduces transcriptional interference with the eukaryotic promoter; excision of CpG-rich backbone sequences limits heterochromatin formation and epigenetic silencing; and vector miniaturization increases the expression cassettes per microgram of administered DNA [15,38]. The resulting increase in the number and longevity of transcriptionally competent templates is particularly advantageous for DNA vaccination, where durable expression of antigen is critical for robust and long-lasting immunity [37].

Conventional plasmids contain immunostimulatory unmethylated CpG motifs that activate pattern recognition receptors such as TLR9 and cytosolic sensors (e.g., cGAS-STING). While this provides intrinsic adjuvanticity, excessive innate activation can impair transfected cell viability, trigger interferon-driven repression of transgene expression, and increase injection site inflammation, thereby limiting repeated dosing [39]. By excising prokaryotic backbone sequences that are rich in unmethylated CpG motifs, mcDNA reduces the structural elements responsible for innate immune recognition. This results in lower inflammatory cytokine induction and reduced reactogenicity compared with conventional plasmid vectors [11]. A representative example of this effect has been demonstrated for an mcDNA vaccine, which showed reduced type I interferon induction and enhanced T-cell immunity compared with its plasmid counterpart (see Section 5.1.3).

The compact size and highly supercoiled topology of mcDNA confer biophysical advantages for non-viral delivery. A smaller hydrodynamic radius enhances complexation with cationic carriers, diffusion through the dense cytosolic environment, and transport to the nuclear envelope [13,40]. These properties increase the percentage of internalized DNA that reaches the nuclear entry site before degradation by nucleases, boosting overall successful transfection efficiency [11,41]. In the context of DNA vaccination, this promotes more efficient transfection of professional antigen-presenting cells, enhancing antigen presentation and downstream priming of both CD4^+^ and CD8^+^ T-cell responses [14].

Conventional plasmids typically carry antibiotic resistance genes for bacterial selection, raising concerns about horizontal transfer of resistance determinants to commensal or pathogenic bacteria [7]. Regulatory guidelines increasingly discourage the use of such markers in clinical vectors or require detailed risk assessments [42]. mcDNA production confines antibiotic selection to the bacterial stage and removes the resistance gene and the origin of replication during recombination, using the ΦC31 integrase or Cre/Xer systems, yielding a backbone-free expression cassette. This design eliminates antibiotic resistance genes from the final product, thereby mitigating the risk of horizontal gene transfer to host microbiota or environmental pathogens [43]. It concurrently reduces the immunostimulatory potential and genomic integration risks associated with residual bacterial sequences. These safety features are particularly important for DNA vaccine applications, where repeated administration, large-scale deployment, and use in healthy populations make the exclusion of resistance determinants and the minimization of vector-related risks essential for clinical acceptance and regulatory approval [42].

While conventional plasmid manufacturing is a well-established, scalable process, mcDNA production is more complex. mcDNA workflows require an additional recombination and purification step to separate minicircles from parental plasmids, backbone circles, and host contaminants. Historically, this has been associated with lower yields and higher costs [12,36]. However, recent innovations in strain engineering, process control, and bacteria-free or cell-free production technologies are improving scalability and cost-effectiveness [44]. These advances are enhancing the commercial viability of mcDNA for vaccine applications and for large-scale vaccine manufacturing. Detailed key features explaining the main differences between mcDNA and conventional plasmid DNA are summarized in Table 1.

## 4. Mechanism of mcDNA Vaccine Action

Safety remains a paramount consideration in the development of DNA vectors for gene therapy and immunization applications. Conventional plasmid DNA vectors carry bacterial sequences, including unmethylated CpG motifs, which can exert context-dependent effects depending on the intended therapeutic application [12]. In gene therapy settings, these motifs activate innate immune sensors such as Toll-like receptor 9 (TLR9) and the cGAS–STING pathway, leading to the production of pro-inflammatory cytokines and type I interferons. This response may promote systemic inflammation and contribute to immune-mediated clearance of transduced cells [12,45]. Consistently, depletion of CpG -rich motifs reduces cytotoxic T-cell responses against vector- or transgene-derived antigens in vivo, underscoring their role in vector immunogenicity [46,47]. In addition, CpG-rich bacterial sequences have been associated with epigenetic silencing via covalent linkage to bacterial DNA elements, causing a reduction in transgene expression over time [12,48].

In contrast, CpG motifs can be beneficial in DNA vaccination, where they function as intrinsic adjuvants that enhance antigen-presenting cell (APC) activation and promote adaptive immune responses [2]. Nevertheless, conventional plasmid vectors also carry antibiotic resistance genes, raising concerns regarding horizontal gene transfer to commensal microbiota [12,49].

mcDNA vectors address these limitations by eliminating bacterial backbone sequences, retaining only the eukaryotic expression cassette. This minimized structure improves biosafety, reduces vector-driven innate activation, and enhances the magnitude and persistence of transgene expression, without altering the core immunological mechanisms of DNA vaccines [12].

**Figure 2:**
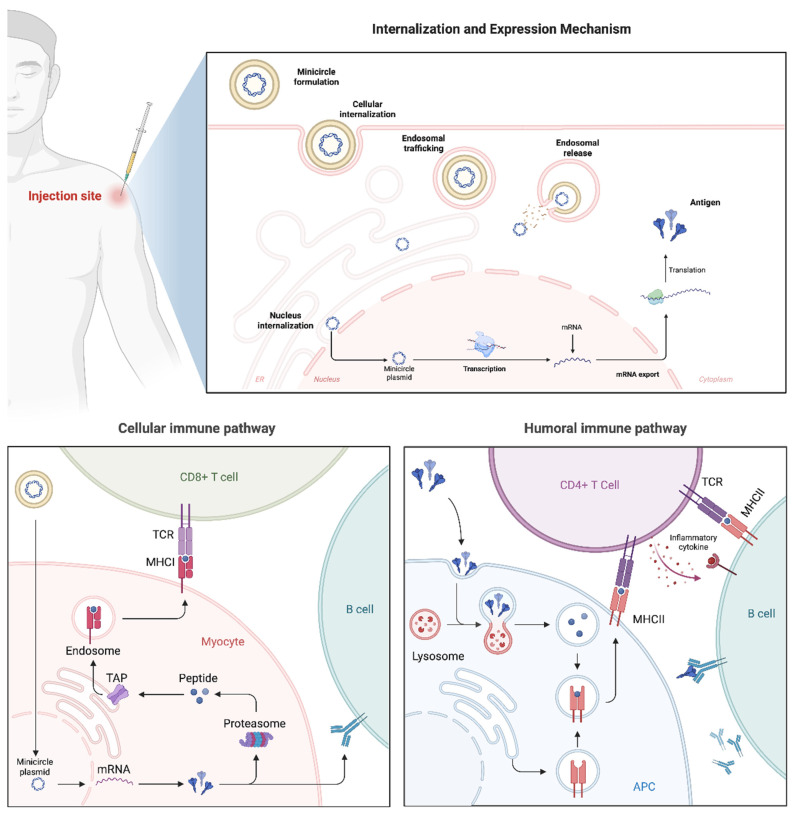
Mechanism of immune activation by mcDNA vaccines. Following intramuscular administration, the mcDNA vaccine formulated with a delivery vehicle is taken up by host cells (myocytes) through endocytosis. Endosomal escape results in the release of mcDNA into the cytoplasm, where it is trafficked to the nucleus. Within the nucleus, the mcDNA remains episomal and is transcribed into mRNA, which is subsequently exported to the cytoplasm, where it is translated into the encoded antigens. These antigens are processed and degraded by proteasomes, producing small peptides that are loaded onto MHC-I molecules and presented on the cell surface, thereby activating CD8^+^ cytotoxic T lymphocytes. APCs can also endocytose the produced antigens and subsequently degrade them in lysosomes, producing small peptides. The peptides get loaded onto MHC II molecules for recognition by CD4^+^ T cells. Activated CD4^+^ T cells provide essential help to B cells, promoting antibody production and the formation of humoral immune memory. Created in BioRender.com. AlFayez, N. (2026) https://BioRender.com/t3nzh03 (accessed on 23 June 2026).

In general, the mechanism of DNA-based vaccines involves several sequential stages: cellular uptake, nuclear delivery, antigen processing, and antigen presentation. The major cellular uptake, intracellular trafficking, transgene expression, and immune activation pathways associated with mcDNA vaccination are summarized in Figure 2.

In professional APCs, such as dendritic cells and macrophages, which are abundant at sites of injection and mucosal surfaces, DNA internalization occurs predominantly via active endocytic mechanisms [50]. These pathways include clathrin-mediated endocytosis, caveolae-mediated endocytosis, caveolae-mediated uptake, and micropinocytosis [51]. This cell-type specificity is immunologically significant because it directly couples DNA internalization to intracellular antigen-processing compartments, favoring MHC-II presentation and cross-presentation through MHC-I, thereby shaping the balance between humoral and cellular immune responses [50,52]. While APCs primarily internalize mcDNA through active endocytic pathways, non-professional cells such as myocytes are thought to internalize DNA primarily through passive membrane diffusion, a process substantially enhanced by physical delivery methods such as electroporation [2,53]. Understanding these cell-specific uptake routes is therefore central to the rational design of delivery systems that direct DNA to the appropriate cellular compartment.

The compact, backbone-free architecture of mcDNA confers practical uptake advantages across both cell types [12]. Joubert et al. demonstrated that mcDNA achieved substantially higher luciferase expression than conventional plasmid DNA under suboptimal electroporation conditions in both fibroblasts and mouse muscle tissue [54]. These findings indicate that mcDNA penetrates the dense extracellular matrix (ECM) more effectively in vitro and in vivo, even when electropermeabilization and electropermeabilization are limited [55].

Following cytoplasmic entry, mcDNA must reach the nucleus to enable transcription, similar to conventional plasmid DNA [56]. In the case of plasmid DNA, nuclear translocation can occur either as free DNA in the cytoplasm or as DNA released from cytoplasmic vesicles [57,58]. Nuclear entry can be mediated through nuclear pores and is facilitated during mitosis, when the nuclear envelope temporarily breaks down [56]. Once inside the nucleus, the DNA is transcribed, and the resulting mRNA is translated in the cytoplasm, ultimately leading to expression of the encoded antigen (Figure 2). However, nuclear entry represents a major rate-limiting barrier: less than 0.1% of internalized plasmid molecules typically undergo productive transcription, reflecting the substantial barrier posed by the nuclear envelope [59]. The reduced size of mcDNA is thought to partially overcome these obstacles by improving nuclear entry [12].

These improvements in nuclear access translate into substantially higher transgene expression in vivo. For example, mcDNA-mediated delivery produced up to a 560-fold increase in circulating human factor IX and α1-antitrypsin levels in the mouse liver following hydrodynamic injection, compared with conventional plasmid DNA [11]. Sustained and enhanced expression has also been demonstrated in stem-cell-derived neural progenitors following microoperation, accompanied by improved cell viability [60]. Together, these findings highlight the capacity of backbone-free mcDNA vectors to enhance both the magnitude and durability of gene expression across diverse target tissues and delivery modalities [12].

Beyond nuclear entry, mcDNA remains episomal and does not integrate into the host genome [12]. This episomal persistence has been demonstrated using Hirt extraction, which selectively enriches extrachromosomal DNA and confirms the presence of mcDNA outside host chromosomes. In addition, fluorescence in situ hybridization (FISH) analysis revealed distinct nuclear signals that did not colocalize with metaphase chromosomes, further supporting the non-integrating nature of mcDNA [61]. mcDNA has also been shown to persist in an episomal state in the liver after hydrodynamic injection in a mouse model of phenylketonuria, indicating stable, long-term expression without detectable genomic integration [62].

The induction of both humoral and cell-mediated immune responses can arise from endogenous or exogenous antigens, depending on the route of administration [56]. DNA-based immunization may involve transfection of somatic cells (e.g., myocytes and keratinocytes) or direct transfection of APCs, as illustrated in Figure 2 [59]. After internalization and expression, multiple pathways contribute to the activation of humoral and cellular immunity [56].

Dendritic cells and macrophages, which are abundant on epithelial and mucosal surfaces, act as key APCs for T-cell and B-cell activation [63]. In the classical endogenous pathways, direct APC transfection (e.g., dendritic cells) enables cytosolic antigen processing and loading of MHC-I peptides, thereby driving CD8^+^ T-cell responses and cytotoxic effector function. Endogenous antigens can also access endosomal compartments for MHC-II presentation and CD4^+^ T-cell activation (Figure 2) [56,64]. In the exogenous pathways, transfected somatic cells release antigens as soluble proteins or apoptotic bodies [56]. APCs take up these antigens for MHC-II presentation to CD4^+^ T cells, promoting B cells to differentiate into antibody-secreting plasma cells (Figure 2) [64]. Importantly, exogenous antigens can also undergo the cross-presentation pathway, whereby APCs process extracellular material and load peptides onto MHC-I molecules, enabling CD8^+^ T-cell priming even without direct APC transfection [56].

Together, these coordinated cellular and immunological mechanisms enable mcDNA vaccines to elicit robust humoral and cellular immune responses. By combining superior transgene expression, epitomal safety, and compatibility with both endogenous and exogenous antigen presentation pathways, mcDNA represents a mechanistically well-founded platform for next-generation immunization.

## 5. Applications of mcDNA in Vaccine Development

Minicircle DNA (mcDNA) has been evaluated across multiple vaccine indications, including infectious diseases and cancer immunotherapy [12]. Its backbone-free architecture, enhanced and durable expression, and favorable safety profile make mcDNA an attractive next-generation platform, particularly when conventional DNA vaccines have shown limited potency.

### 5.1. Infectious Disease Vaccines

#### 5.1.1. Bacterial and Parasitic Infections

Conventional plasmid DNA vaccines are often limited by short-lived transgene expression due to transcriptional repression driven by bacterial backbone sequences, which limits the magnitude and duration of protective immunity [14,42]. mcDNA constructs, by contrast, have been tested in various bacterial and parasitic diseases, showing stronger and more sustained expression, which can translate into improved protection. Regarding eradicating bacterial infection, it has been shown that intradermal administration of mcDNA encoding a SIINFEKL-containing antigen expression cassette induced prolonged antigen expression, vigorous antigen-specific CD8^+^ T-cell responses, and substantial protective immunity against *Listeria monocytogenes* infection in mice compared with a similar traditional plasmid DNA vaccine. In addition, parasitic models and mcDNA-based vaccines generated higher antibody titers and more robust T-cell responses, underscoring their ability to enhance immunity against intracellular pathogens [14]. Although bacterial and parasitic mcDNA vaccines show encouraging early results, their transition to clinical use is currently limited by the need to achieve durable protection across various pathogens and optimize delivery efficacy, safety, and administration devices [65].

Beyond expression, the removal of antibiotic-resistance genes from mcDNA also has practical regulatory significance, as detailed in Section 3; this backbone minimization step is best understood as proactive risk mitigation rather than a response to an established clinical hazard [12,66]. Moreover, independent groups have corroborated these advantages across additional vaccine indications. A minicircle-based vaccine against the hepatitis C virus elicited potent antigen-specific T-cell and antibody responses superior to those of its parental plasmid, and an antibiotic-resistance-gene-free minimized DNA vaccine (asd-pVAX1) likewise produced enhanced immunogenicity relative to a conventional plasmid control [67,68]. These independent reports broaden the evidence base for backbone-minimized DNA vaccines beyond any single laboratory.

#### 5.1.2. Hepatitis C Virus (HCV)

The HCV poses a significant global health challenge due to its genetic diversity and immune evasion mechanisms, which hinder vaccine efficacy. Traditional approaches that rely solely on humoral or T-cell responses have struggled to achieve broad, durable protection. mcDNA vaccines encoding conserved HCV epitopes, for example, constructs combining an HBsAg-linked E2 epitope (sHBsAG_412–425_) with HCV nonstructural proteins, have been shown to effectively stimulate both humoral and cellular immunity, eliciting broadly reactive antibodies and strong multi-epitope HCV-specific T-cell responses. Overall, these findings suggest that mcDNA can support balanced humoral cellular immunity, while accommodating complex, multigenic antigen designs are needed for a pan-genotypic HCV vaccine [67]. The constructs encoding HCV antigens can elicit T-cell and antibody responses, supporting the platform’s feasibility. However, issues such as broad genotype coverage, long-term immunity, and model validation remain unresolved [67]. Of the infectious disease indications reviewed here, the HCV faces the steepest translational barriers, as its extreme genetic diversity demands a pan-genotypic antigen design not yet achieved with any platform, and the lack of a validated small-animal model limits preclinical-to-clinical extrapolation [69,70,71].

#### 5.1.3. Human Immunodeficiency Virus Type 1 (HIV-1)

HIV-1 remains one of the challenging targets for DNA vaccination, as conventional plasmid vectors often elicit low antigen expression and weak, transient CD8^+^ T-cell responses in vivo, especially in large-animal models and humans. As a result, despite strong theoretical advantages, DNA vaccine platforms have not yet achieved sufficient efficacy for clinical translation in HIV settings [64]. Wang et al. directly addressed this limitation by comparing an mcDNA HIV-1 vaccine encoding a codon-optimized *gag* gene with its parental pVAX1-gag plasmid, both delivered intramuscularly by in vivo electroporation. The mcDNA gag constructs achieved significantly higher intramuscular antigen expression, around 60% lower serum IFN-α levels, and about two-fold higher Gag protein expression than the matched plasmid at the same DNA dose. These expression gains translated into more robust and durable HIV-specific CD8^+^ T-cell responses, including increased percentages of IFN-γ-producing T cells and stronger cytolytic activity, while maintaining an acceptable local reactogenicity profile. Interestingly, mcDNA also improved dose efficiency, achieving comparable or superior responses at reduced DNA doses relative to pVAX1-gag [64]. Collectively, this example demonstrates how backbone-free mcDNA vectors can overcome key bottlenecks of plasmid DNA in HIV vaccination by decoupling antigen expression from excessive innate immune activation and enhancing both the magnitude and quality of antiviral T-cell responses [72]. Nevertheless, the absence of a defined correlate of protection remains a fundamental obstacle: no T-cell response parameter has reliably predicted clinical efficacy in humans, as illustrated by the STEP trial and HVTN 702, both of which generated robust T-cell responses yet conferred no protection against HIV acquisition [73,74]. For DNA-based platforms, heterologous prime-boost regimens consistently outperform DNA vaccination alone in non-human primate models [75,76]. mcDNA’s most realistic near-term contribution is therefore as a priming component within such strategies, leveraging its sustained antigen expression to reinforce initial immune priming by mRNA or viral vectors, rather than as a standalone vaccine, pending direct head-to-head comparisons with conventional plasmid DNA in NHP models [72].

#### 5.1.4. Severe Acute Respiratory Syndrome Coronavirus 2 (SARS-CoV-2)

COVID-19 is a highly contagious respiratory illness caused by the SARS-CoV-2 virus, which first emerged in late 2019, sparking a global pandemic and evolving into a persistent health issue that requires widespread vaccination and medical advancements [64]. The first authorized COVID-19 vaccines used mRNA-based formulations (such as mRNA-1273) encoding the spike (S) protein, demonstrating, for the first time at scale, the efficacy, safety, and manufacturability of nucleic acid vaccines [64]. However, DNA vaccines offer comparable conceptual advantages with greater stability and potentially simpler manufacturing and distribution [51]. mcDNA vectors encoding SARS-CoV-2 antigens, such as the receptor-binding domain (RBD) of the spike protein, have been investigated using advanced delivery systems. For example, Dalinda Eusébio et al. employed mannosylated, cholesterol-functionalized polyethylenimine (PEI) nanocarriers to deliver both plasmids and mcDNA RBD constructs to APCs; mcDNA formulations achieved superior cellular uptake, higher gene and protein expression, and strong dendritic cell activation. These data support the use of mcDNA as a flexible platform for rapid-response vaccines against emerging viral pathogens [64]. SARS-CoV-2 offers a more feasible opportunity for mcDNA translation than many chronic viruses because of clear antigen targets, immune responses, and development pathways. However, for clinical use, mcDNA must outperform mRNA and protein vaccines in stability, dose efficiency, durability, safety, or cost [22,77].

### 5.2. Cancer Vaccines and Immunotherapy

Although many DNA cancer vaccines still use conventional plasmid vectors, mcDNA is increasingly recognized as an advantageous alternative for encoding tumor-associated antigens and personalized neoepitopes [78,79]. DNA cancer vaccines aim to induce potent CD8^+^ cytotoxic and CD4^+^ helper T-cell responses against tumor antigens, and preclinical studies have shown that neoantigen-based DNA vaccines can delay and control tumor growth in murine models [79,80,81]. mcDNA’s compact size and backbone-free design facilitate the construction of multi-epitope and polyepitope expression cassettes, supporting prolonged, high-level expression of multiple tumor antigens from a single vector. These characteristics facilitate robust cellular immunological responses and position mcDNA as a compatible platform for combinatorial approaches incorporating immune checkpoint inhibitors and other immunomodulatory medicines [12,78,82].

In several preclinical tumor models, mcDNA encoding tumor-associated antigens or neoepitopes has induced stronger neoantigen-specific T-cell responses and superior tumor control compared with conventional plasmid DNA vaccines [14]. By contrast, combining conventional plasmid DNA-encoded neoepitopes with immune checkpoint inhibitors, such as anti-PD-1, has been shown to enable effective tumor control with enhanced CD4^+^ and CD8^+^ effector responses and improved survival outcomes [80]. Although this work used a plasmid rather than mcDNA, it provides a conceptual and translational template for extending neoepitope–checkpoint inhibitor combination strategies to mcDNA-based platforms, which would be expected to achieve comparable or superior effects at lower doses given mcDNA’s enhanced expression profile [14,64]. Collectively, the higher transgene expression and cleaner vector backbone of mcDNA may enable dose reductions and improved safety margins, particularly within personalized neoantigen vaccine pipelines or when deployed alongside standard-of-care checkpoint inhibitors and other immunotherapy combinations. Cancer vaccines suit mcDNA due to sustained antigen expression, multi-epitope designs, and dose-sparing delivery, which are vital for neoantigen and tumor vaccination. Challenges include tumor heterogeneity, immunosuppression, and delivery optimization [83].

Recent advances in plasmid DNA vaccine technology reinforce this trajectory: optimized plasmid platforms combined with checkpoint blockade have now entered the clinic, providing a direct benchmark for backbone-minimized vectors such as mcDNA. In a representative example, the personalized DNA neoantigen vaccine GNOS-PV02, delivered via electroporation and combined with the anti-PD-1 antibody pembrolizumab in advanced hepatocellular carcinoma, was well tolerated and induced both CD8^+^ cytotoxic and CD4^+^ helper T-cell responses specific for the encoded neoantigens, with an objective response rate and survival outcomes that compared favorably with historical anti-PD-1 monotherapy in this setting. Because mcDNA can achieve equivalent or greater antigen expression at lower doses than conventional plasmids, these findings support the rationale for deploying mcDNA-encoded neoantigens alongside anti-PD-1 checkpoint inhibitors to enhance CD4^+^/CD8^+^ effector responses and improve survival [84].

Moreover, early-stage preclinical studies have begun to explore the feasibility of mcDNA as a target for a specific cancer vaccine. As discussed above, Dietz et al. demonstrated the immunological superiority of mcDNA over conventional plasmid DNA in eliciting antigen-specific CD8^+^ T-cell responses, supporting the broader applicability of mcDNA as a cancer platform. Building on this, Almeida et al. demonstrated the feasibility of producing a regulatory-grade supercoiled mcDNA vaccine encoding the HPV *E7* oncoprotein, confirming successful antigen expression via immunocytochemistry and establishing a robust size-exclusion chromatography purification workflow that meets regulatory standards [85]. While this study focused on production methodology and expression assessments rather than in vivo immunogenicity, it represents an important step toward developing a manufacturable, clinically translatable mcDNA platform for a therapeutic vaccine. It provides the production framework needed for future preclinical evaluation.

Despite these encouraging results, a balanced appraisal of the vaccine applications above must be performed to weigh genuine translational potential against concrete barriers to clinical adoption. The clearest near-term opportunity lies in therapeutic cancer vaccination, yet several obstacles remain specific to mcDNA. First, the additional intramolecular recombination and purification steps that confer mcDNA’s safety advantages also reduce manufacturing yield and raise the cost of goods and remain insufficiently standardized for industrial-scale GMP production. Second, efficient and reproducible in vivo delivery is still the dominant efficacy bottleneck, and no consensus on the route–formulation combination has yet emerged. Third, the clinical evidence base remains dominated by small, single-arm studies that use conventional plasmids rather than mcDNA, so head-to-head data isolating the contribution of backbone minimization in humans is largely absent. Finally, the regulatory pathway for mcDNA is comparatively immature, with mcDNA-specific comparability criteria, potency assays, and long-term safety expectations not yet harmonized. Realizing mcDNA’s promise in these vaccine applications will therefore depend less on further proof of biological superiority, which is already substantial, than on de-risking manufacturing, delivery, and regulatory translation [84,86].

## 6. Delivery Strategies for mcDNA Vaccines

Efficient delivery remains a critical determinant of DNA vaccine immunogenicity [87]. Although mcDNA intrinsically improves transcriptional activity and persistence, its performance remains highly dependent on the delivery platform. Because mcDNA vectors are typically 30–60% smaller than their parental plasmids, vector miniaturization increases the gene copy number per microgram of DNA and alleviates several size-related delivery constraints [12]. For example, a hepatitis B vaccine construct was reduced from 5737 bp in plasmid pCMV-S2S to 3153 bp in minicircle MC07.CMV-HBS2S, theoretically enabling a ~1.82-fold increase in expression cassette copy number per microgram of DNA due to vector size reduction [88]. This reduction in size also facilitates more efficient cellular uptake, nuclear entry, and dose-sparing antigen expression [89].

### 6.1. Physical Delivery Methods

#### 6.1.1. Electroporation

Conventional plasmid DNA vaccines often require electroporation to overcome inefficient cellular uptake and transcriptional silencing [90]. mcDNA partly mitigates this dependence by preserving vector integrity and transcriptional competence. Under comparable electroporation conditions, mcDNA shows greater uptake and more sustained tissue expression than full-length plasmids, achieving equivalent transgene levels at lower field strengths or with fewer pulses [89]. In needle-free pneumatic delivery, plasmid DNA is more susceptible to shear-induced nicking, whereas mcDNA better retains its supercoiled conformation and exhibits a markedly lower proportion of nicked species [42]. Across multiple electroporation studies, mcDNA has produced approximately 2.4- to 10.8-fold higher expression in murine muscle and airway models, along with prolonged expression kinetics [37,91]. Importantly, mcDNA enables effective transfection at reduced electrical field strengths, allowing expression comparable to that of plasmid DNA delivered at 100–120 V/mm, which was achieved with 60–80 V/mm pulses, thereby improving tolerability and reducing tissue injury and inflammation [87,92].

#### 6.1.2. Gene Gun Delivery

A gene gun, also known as particle-mediated epidermal delivery, propels DNA-coated microcarriers (typically gold or tungsten) into the epidermis and dermis using high-pressure devices [4,93]. This approach enables direct intracellular delivery to keratinocytes and skin-resident antigen-presenting cells and has demonstrated superior immunogenicity compared with intramuscular injection in several models [94]. Gene gun administration supports pronounced dose sparing, with robust immune responses elicited using nanogram-scale DNA doses. The gene gun also permits simultaneous delivery of multiple genetic constructs to the same tissue, facilitating the analysis of interactions among co-expressed gene products [95]. mcDNA vectors can further enhance gene gun performance by producing higher and longer-lasting transgene expression than plasmid DNA, thereby increasing expression efficiency per unit dose and supporting low-dose DNA vaccination strategies [96]. However, a primary constraint remains its shallow tissue penetration, which is limited to the outermost 100 to 500 μm of the tissue, thereby restricting its biomedical and experimental applications predominantly to the cutaneous layers, superficial musculature, or surgically exposed ex vivo and in vitro tissue systems [97].

### 6.2. Chemical Delivery Systems

Chemical delivery platforms are attractive for clinical translation because they avoid the invasiveness and infrastructure requirements of physical methods. The reduced size and optimized topology of mcDNA provide broad advantages across non-viral carriers, including improved condensation, enhanced cellular uptake, lower carrier requirements, and superior tolerability relative to plasmid DNA [95].

#### 6.2.1. Lipid Nanoparticles (LNPs)

LNPs represent the most clinically advanced non-viral delivery system. Although developed primarily for mRNA, mcDNA exhibits favorable structural properties for lipid encapsulation, including a smaller size and improved homogeneity [98,99]. mcDNA-loaded LNPs generally show higher encapsulation efficiency, a smaller particle size, and lower polydispersity than equivalent plasmid formulations, enabling effective delivery at reduced lipid-to-DNA ratios [99,100]. In liver-targeted preclinical models, mcDNA-loaded LNPs have achieved higher transgene expression with improved tolerability, consistent with a reduced lipid burden [101]. Despite their clinical success, standard LNPs remain limited for minicircle DNA (mcDNA) delivery because of structural packing challenges, which are driven by the rigidity and high charge density of double-stranded DNA, and a natural hepatic tropism that restricts efficient extrahepatic targeting without complex surface modification [77,102].

#### 6.2.2. Cationic Polymers and Other Non-Lipid Nanocarriers

mcDNA also performs well in polymer-based systems such as PEI, poly(L-lysine), and sequence-defined oligoaminoamides [103]. These polymers condense mcDNA into smaller, more homogeneous polyplexes, often <80 nm, compared with complexes formed with plasmid DNA at matched charge ratios [103]. This improved compaction enables several-fold higher expression at lower nitrogen-to-phosphate ratios, accompanied by substantial reductions in polymer-associated cytotoxicity and better tissue compatibility in vivo [104]. Similar benefits are observed in niosomes, polymeric micelles, and polysaccharide-based carriers, in which mcDNA provides more persistent expression and reduced inflammatory signaling compared with plasmid DNA [105]. Although cationic polymers and non-lipid systems enhance minicircle DNA compaction, their therapeutic utility remains limited by charge-mediated serum aggregation and nuclease sensitivity, as well as intracellular unpacking failures and cumulative, non-biodegradable cytotoxicity [106,107].

### 6.3. Comparative Evaluation of Efficiency, Toxicity, and Translational Feasibility

Across delivery modalities, mcDNA consistently exhibits a more favorable efficiency-to-toxicity ratio than conventional plasmid DNA [95]. Physical delivery methods, particularly electroporation, produce the highest absolute expression levels, with mcDNA routinely achieving 3- to 10-fold higher peak expression and prolonged persistence in preclinical models [99,100]. However, electroporation-related discomfort and tissue damage remain limiting factors for widespread prophylactic use [98]. Chemical systems achieve lower peak expression but benefit disproportionately from mcDNA payloads: LNP-based formulations commonly show 2- to 5-fold higher expression at matched lipid doses, while polymer-based carriers achieve 3- to 10-fold gains at reduced carrier concentrations [108].

Consistent with these trends, a nuclear-targeted electroporation study demonstrated that mcDNA encoding human ANGPT1 produced 2048 ± 567 pg/mL, which is about 3.7-fold higher secreted protein levels at 24 h, compared with 552.1 ± 33.5 pg/mL for plasmid DNA under identical delivery conditions, indicating intrinsic transcriptional superiority rather than differences in transfection efficiency or cytotoxicity [11,92]. This expression advantage is not restricted to high-energy physical delivery and extends to low-energy chemical systems. Similarly, in c-Met-targeted, polyethylene glycol (PEG)-shielded polyplexes, mcDNA encoding luciferase formed nanoparticles of approximately 35–40 nm, versus 65–100 nm for plasmid complexes, and achieved up to ~200-fold higher gene transfer efficiency in c-Met-positive prostate cancer cells, underscoring the delivery-agnostic performance benefits conferred by miniaturization and topology optimization [109].

Overall, the evidence indicated that mcDNA improves the performance of both physical and chemical delivery systems by combining compact size with enhanced transcriptional competence. While physical methods remain highly effective for maximizing expression, chemical carriers are likely to offer greater translational flexibility, particularly when paired with mcDNA to reduce payload burden, improve tolerability, and support scalable vaccine deployment (Table 2).

## 7. Advantages and Limitations of mcDNA Vaccines

mcDNA vaccines represent an advanced plasmid-derived platform engineered to overcome key limitations of conventional plasmid DNA [110]. By eliminating bacterial backbone elements, such as antibiotic resistance genes and origins of replication, mcDNA offers improved safety, enhanced and more sustained transgene expression, and superior immunogenicity [12]. Nevertheless, important technical, manufacturing, and regulatory challenges must be resolved before their broad clinical implementation.

From a safety perspective, mcDNA’s backbone-free design eliminates antibiotic resistance genes and bacterial CpG motifs from the final product (as detailed in Section 3) [12,87,111]. Its episomal element further reduces the risk of insertional mutagenesis [12]. At the same time, reduced epigenetic silencing supports prolonged, higher-level transgene expression in vivo [6]. However, despite its improved biological stability, the physical stability of naked mcDNA remains susceptible to nuclease degradation and requires appropriate formulations, delivery systems, and storage conditions to ensure long-term stability and effective in vivo performance [112].

In terms of immunogenicity, mcDNA vaccines demonstrated robust humoral and cellular immune responses across multiple disease models, including viral and bacterial infections, parasitic diseases, cancer, and genetic disorders [113]. Enhanced and sustained antigen expression translates into higher antigen-specific antibody titers, stronger CD8^+^ cytotoxic and CD4^+^ helper T-cell responses, and, in many settings, improved protection or tumor control compared with conventional plasmid DNA vaccines [37,114].

In cancer vaccine models, mcDNA encoding tumor-associated antigens or neoepitopes can elicit potent cytotoxic T lymphocyte responses, leading to improved tumor control and survival, particularly when combined with immune checkpoint inhibitors [78]. Furthermore, mcDNA often achieves these effects at lower doses than plasmid vectors, which may reduce toxicity, simplify manufacturing and logistics, and improve patient acceptability [67,115].

Despite these advantages, several limitations remain. mcDNA is more complex than classical plasmid manufacturing, typically requiring an additional recombination step and more stringent purification to remove residual parental plasmids, excised backbones, and host-derived contaminants [36]. These added steps can negatively impact production yield and increase manufacturing costs, particularly at a large scale. Bacteria-free and cell-free synthesis platforms are beginning to address these issues, but they are not yet widely standardized at an industrial scale [24,116].

Additionally, although preclinical data strongly support mcDNA’s superior performance, efficient in vivo delivery remains a critical bottleneck for mcDNA vaccines [89].

Beyond delivery efficiency, several translational barriers continue to limit the clinical adoption of mcDNA vaccines. One major challenge is the lack of large-scale clinical validation. While several preclinical studies have demonstrated superior transgene expression and immunogenicity compared with conventional plasmid DNA, evidence from human clinical trials remains limited [117]. Consequently, the extent to which these preclinical advantages translate into meaningful clinical efficacy across different patient populations is still uncertain.

Another important consideration is the absence of standardized manufacturing and quality-control frameworks specific to mcDNA products. Regulatory agencies require comprehensive characterization of vector identity, purity, potency, residual parental content, and long-term stability. However, consistent analytical standards and release criteria for mcDNA are not yet fully established, creating additional complexity for regulatory approval and technology transfer between manufacturing sites [118,119].

Economic and logistical factors may also influence the clinical adoption of mcDNA vaccines. Although mcDNA can potentially achieve therapeutic efficacy at lower doses, the specialized production processes, advanced purification requirements, and formulation technologies may initially increase manufacturing costs compared with conventional plasmid platforms. Furthermore, large-scale commercialization will require robust supply chains, validated storage conditions, and reproducible batch-to-batch consistency that comply with current Good Manufacturing Practice (cGMP) standards [87].

Clinical implementation may be further complicated by the need for optimized delivery technologies. Many DNA vaccine platforms rely on electroporation or nanoparticle-based delivery systems to achieve sufficient cellular uptake, which can increase procedural complexity, cost, and variability across clinical settings. The co-development and regulatory evaluation of both the mcDNA construct and its associated delivery system may therefore prolong development timelines and increase translational risk [4,90].

While mcDNA exhibits a favorable theoretical safety profile owing to its episomal nature and lack of bacterial backbone sequences, extensive post-administration follow-up will be required to establish long-term persistence, biodistribution, immunogenicity, and rare adverse-event profiles in humans [120]. Addressing these scientific, manufacturing, economic, and regulatory challenges will be critical for the successful translation of mcDNA vaccines from experimental platforms to routine clinical use.

Currently, most clinical DNA vaccine and gene therapy trials still use conventional plasmids. Regulatory agencies are familiar with plasmid-based vectors, whereas mcDNA-specific guidance, comparability criteria, and long-term safety datasets are still emerging.

Overall, mcDNA vaccines represent a promising next-generation DNA vaccine platform with clear advantages in safety, immunogenicity, and transgene expression. However, challenges related to scalable manufacturing, formulation, and clinical evaluation must be addressed to realize their translational potential fully. Continued advances in vector engineering, delivery technologies, and regulatory standardization are expected to accelerate the translation of mcDNA vaccines into clinical practice. The main advantages and limitations of mcDNA as a possible DNA-based vaccine are summarized in Table 3.

## 8. Future Perspectives and Recommendations

Although the preclinical evidence reviewed above strongly supports mcDNA as a next-generation non-viral vector platform, several technical, manufacturing, and regulatory barriers must be addressed before widespread clinical deployment becomes feasible. Future progress will depend on advances in scalable production, optimized delivery systems, and robust clinically relevant models and human studies.

### 8.1. Production and Scalability

At present, bacterial production systems, such as engineered *E. coli* strains, including ZYCY10P3S2T, remain the most widely used approach for mcDNA manufacturing. However, these workflows are often associated with long processing times, residual endotoxin contamination, and incomplete recombination, which can leave 3–15% of the preparation as parental plasmid or backbone circle contamination [20]. These limitations may reduce yield, compromise batch consistency, and raise regulatory concerns, thereby constraining large-scale vaccine manufacturing.

To address these obstacles, emerging technologies have been proposed to improve production, including cell-free in vitro methods that may improve purity, shorten turnaround time, and eliminate bacterial contaminants. Among the most promising are the bacteria-free (BF) method, the plasmid2MC workflow, and the Circular Vector (CV) approach (described in detail in Section 2). Collectively, these platforms offer cleaner production routes and may reduce the risk of endotoxin-related toxicity or loss of vector activity during delivery [24,30,121]. Importantly, some methods have demonstrated higher transgene expression and improve cell viability compared with traditional bacterial systems, suggesting that manufacturing improvements may translate into biological performance [24,30]. Nevertheless, further validation under GMP-compliant conditions will be essential, particularly with respect to batch-to-batch consistency and scalability to industrially relevant yields.

### 8.2. Delivery Optimization

Efficient in vivo delivery remains one of the most significant determinants of mcDNA vaccine performance. While mcDNA’s compact size and optimized topology confer intrinsic advantages in cellular uptake and nuclear entry, as discussed in Section 3, the choice of delivery platform remains critical for achieving sufficient immunogenicity in vivo. Future efforts should prioritize delivery systems that improve targeting to APCs while reducing the infrastructure demands associated with current physical delivery methods.

LNPs have emerged as a promising delivery candidate for the clinical translation of mcDNA vaccines, given their established regulatory precedent from mRNA vaccine development and their demonstrated compatibility with mcDNA payloads across a range of applications, including vaccines for cancer and infectious diseases, gene therapy, and gene editing for genetic disorders [122,123,124]. Further optimization of ionizable lipid composition and surface functionalization, particularly mannosylation for APC targeting, may enhance transfection efficiency and immunogenicity at lower doses, thereby reducing systemic toxicity [125,126,127].

Exosome-based delivery systems and polymeric nanocarriers, on the other hand, have been proposed as efficient and safe vehicles for nucleic acids, shuttling DNA and RNA between cells [128,129]. These platforms may offer complementary avenues, particularly for tissue-specific targeting, though further preclinical validation is required prior to clinical translation [130].

Beyond chemical delivery systems, physical delivery methods such as electroporation continue to achieve effective gene delivery in preclinical models, but their invasiveness and equipment requirements limit their practicality for large-scale prophylactic vaccination [131]. Emerging needle-free and microneedle-based approaches may address these limitations by reducing procedural complexity while maintaining immunogenicity [132].

### 8.3. Clinical Translation and Future Vaccine Strategies

mcDNA is now transitioning from a proof of concept to early clinical application, supported by a growing body of preclinical literature and a small but expanding set of clinical programs [12,99,133]. Pioneering examples include CARAMBA, which uses mcDNA-based constructs to generate virus-free CAR-T cells for multiple myeloma and has progressed to Phase I/II evaluation [134]. While this program focuses on cell therapy rather than direct vaccination, it provides important evidence that mcDNA-based platforms can be manufactured under GMP conditions and translated into clinical settings, providing a regulatory and technological framework relevant to future mcDNA vaccine development.

In parallel, mRNA–lipid nanoparticle (mRNA-LNP) vaccines have emerged as a transformative nucleic acid delivery platform, characterized by strong immunogenicity, a favorable safety profile due to their non-integrative nature, and rapid, scalable, and manufacturing capabilities [135]. The LNP component protects mRNA from nuclease degradation and facilitates efficient cellular uptake and endosomal escape, enabling cytoplasmic translation of the encoded antigen and rapid induction of both humoral and cellular immune responses [136,137]. These attributes enabled the accelerated clinical translation and emergency authorization of mRNA vaccines during the COVID-19 pandemic, demonstrating their suitability for widespread human use [138,139].

Compared with mRNA-LNP systems, DNA-based platforms, including mcDNA, require nuclear entry prior to transcription, which can delay antigen expression. However, DNA vectors may support more sustained antigen production and longer-lasting immune responses [140]. In particular, mcDNA combines the durability of DNA platforms with improved safety and expression efficiency due to its minimized, backbone-free design [15,37]. In contrast, mRNA-driven immunity, while rapid, is often limited by transient antigen expression and declining antibody titers over time [141,142].

These differences suggest that mcDNA-based vaccines may complement or, in certain contexts, provide an alternative to mRNA platforms, particularly in applications requiring prolonged antigen exposure and durable immunity, such as chronic infections, cancer immunotherapy, and therapeutic vaccination [14,64,143].

Looking forward, nucleic acid vaccine development is likely to shift toward integrated and complementary strategies rather than platform competition [144]. Heterologous prime-boost regimens combining mRNA-LNP and mcDNA could exploit the rapid antigen expression of mRNA for initial immune priming and the sustained expression of mcDNA for reinforcement and long-term durability [145,146]. In addition, advances in delivery technologies, including LNP formulations adapted for DNA carrier systems and targeted nanoparticles, may further improve nuclear delivery efficiency while preserving the stability advantages of mcDNA [147,148].

### 8.4. Remaining Challenges and Future Priorities

Before clinical translation of mcDNA vaccines, several priorities must be addressed. First, GMP-compatible production workflows must be reproducible, scalable, and cost-effective [149]. Second, direct head-to-head comparisons of delivery platforms should be performed across relevant vaccine indications to identify the most effective route–formulation combinations [64]. Third, immunogenicity and durability should be validated in large-animal models that better reflect human anatomy, dosing constraints, and immune responses [21]. Finally, regulatory harmonization will be essential, including standardized quality-control frameworks for mcDNA purity, residual parental plasmid content, and recombination efficiency [149].

Overall, mcDNA represents a highly promising evolution of DNA vaccine technology, with a strong mechanistic rationale and substantial preclinical support. Its clinical success will depend on the convergence of improved manufacturing, smarter delivery systems, and rigorous translational validation. If these challenges are addressed systematically, mcDNA could emerge as a versatile and clinically competitive platform for both prophylactic and therapeutic cancer immunization and non-viral gene delivery.

## 9. Conclusions

mcDNA has emerged as a compelling next-generation platform for DNA vaccine development, offering structural, immunological, and biosafety advantages that directly address several limitations of conventional plasmid-based approaches. By eliminating bacterial backbone sequences, including antibiotic resistance genes, origins of replication, and CpG motifs, mcDNA can achieve a more favorable safety profile, higher and more sustained transgene expression, and reduced vector-driven innate immune activation. Preclinical evidence from infectious disease and cancer vaccine models consistently shows that these properties translate into stronger antigen-specific CD8^+^ cell responses, enhanced humoral immunity, and improved therapeutic outcomes compared with matched plasmid DNA vaccines.

Despite this encouraging foundation, the clinical deployment of mcDNA vaccines remains at an early stage. Manufacturing complexity, lower production yields relative to conventional plasmids, and dependence on specialized delivery systems are all limitations. Addressing these challenges will require coordinated advances in GMP-compatible and cell-free production workflows, such as systematic optimization of delivery platforms, particularly LNPs and targeted polymeric carriers, for specific vaccine indications and the generation of robust large-animal and early-phase human efficacy data to bridge the gap between preclinical promise and clinical reality. Importantly, the clinical advancement of CARAMBA, which employs an mcDNA-based construct to generate virus-free CAR-T cells, shows that mcDNA-based therapies can be translated into human trials. With these advances, mcDNA is well-positioned to become an important next-generation nucleic acid-based vaccine and immunotherapy, particularly in applications where dose efficiency, optimal antigen expression, and a favorable biosafety profile are critical, such as therapeutic cancer vaccines and rapid-response platforms for emerging infectious diseases.

## Figures and Tables

**Table 1:** Summary of the key features of mcDNA versus conventional plasmid DNA.

Feature	Minicircle DNA (mcDNA)	Conventional Plasmid DNA (pDNA)
**Molecular Size**	Small; contains only the eukaryotic expression cassette.	Larger; includes a prokaryotic backbone (origin of replication, antibiotic resistance gene, and bacterial sequences).
**Transgene Expression**	Persistent and high-level; resistant to epigenetic silencing.	Lower and less sustained; susceptible to rapid silencing.
**Innate Immune Activation**	Reduced; lacks immunostimulatory unmethylated CpG motifs, leading to lower inflammatory cytokine induction and less reactogenicity.	High; rich in unmethylated CpG motifs that activate TLR9/cGAS-STING pathways, causing inflammation and potentially suppressing antigen expression.
**Cellular Uptake and Nuclear Delivery**	Improved due to a smaller hydrodynamic radius, enhancing complexation with delivery agents and cytosolic/nuclear transport.	Less efficient due to the larger size, hindering diffusion and nuclear entry.
**Safety Profile**	Superior; no antibiotic resistance gene in the final product, eliminating the risk of horizontal gene transfer and reducing genomic integration risks.	Concerns: contains antibiotic resistance markers, raising issues of horizontal gene transfer and contributing to antimicrobial resistance concerns.
**Manufacturing**	More complex; it involves recombination and stringent purification. Historically, it has a lower yield/cost, but new methods (such as cell-free synthesis) are improving scalability.	Well-established, scalable fermentation process. Simpler and typically more cost-effective.

**Table 2:** Comparative matrix of mcDNA delivery platforms.

Delivery Platform	Delivery	Specificity	Expression	Feasibility
Electroporation	mcDNA is administered and electrically transferred into local tissue but may require lower electrical field strengths than plasmid DNA to achieve comparable expression.	Low intrinsic APC specificity; mainly transfects local muscle or epithelial cells, followed by antigen release, APC uptake, and cross-presentation.	mcDNA can produce ~ 2.4- to 10.8-fold higher expression in murine muscle and airway models; for example, human ANGPT1 produced 2048 ± 567 pg/mL, which is about 3.7-fold higher than that produced by plasmid DNA.	Efficient for local delivery but limited by discomfort, tissue injury, inflammation, specialized equipment, and weak APC targeting. These may restrict broader use, though it remains a top delivery method for therapeutic mcDNA vaccination [37,42,87,89,90,91,92].
Gene gun/particle-mediated epidermal delivery	DNA is adsorbed onto gold or tungsten microcarriers rather than encapsulated, supporting dose-sparing delivery, often at nanogram-scale DNA doses.	Moderate; delivery to epidermal and dermal compartments can expose DNA to keratinocytes, Langerhans cells, and skin-resident dendritic cells.	Supports localized DNA vaccination and simultaneous delivery of multiple constructs. mcDNA improves expression efficiency per unit dose compared with plasmid DNA.	Localized and dose-sparing but constrained by device requirements, particle dose, variable tissue penetration, and limited scalability. It remains useful for skin-directed vaccination, but mcDNA-specific evidence is less developed than for electroporation [4,93,94,95,96].
Lipid nanoparticles/lipid-based carriers	Formulation-dependent. mcDNA’s smaller size and improved homogeneity may support higher encapsulation efficiency, a smaller particle size, lower PDI, and reduced lipid-to-DNA ratios compared with larger plasmids.	Low without ligand modification; APC specificity requires surface engineering, such as mannose, peptides, antibodies, or other ligand decorations.	mcDNA-LNP systems can achieve higher transgene expression and improved tolerability in liver-targeted preclinical models.	Lipid-based systems are widely used as non-viral nucleic acid carriers and are highly scalable. However, details on mcDNA-specific vaccines’ biodistribution, nuclear delivery, reactogenicity, and APC-targeting capabilities are still unclear [98,99,100,101].
Polymers and non-lipid nanocarriers, including PEI, poly-L-lysine, and oligoaminoamides	High electrostatic condensation capacity. mcDNA can form smaller and more homogeneous polyplexes, often with reduced sizes, compared with plasmid DNA at matched charge ratios.	Low unless ligand-modified; APC specificity can be improved by mannose, peptides, or receptor-targeting ligands.	mcDNA polyplexes can produce several-fold higher expression at lower nitrogen-to-phosphate ratios, with reduced carrier requirements compared with plasmid DNA.	These systems are flexible for APC-targeted mcDNA delivery, but cationic charge can cause cytotoxicity, serum instability, aggregation, and inflammation. Shielding and reduced carrier use may improve tolerability, but reproducibility and in vivo validation remain major barriers [103,104,105].
Targeted polyplexes	In a c-Met-targeted PEG-shielded polyplex model, mcDNA formed particles of approximately ~40 nm, compared with 2× the size for plasmid complexes.	Receptor-specific but not necessarily APC-specific. APC targeting would require ligands directed to dendritic-cell or macrophage receptors.	c-Met-targeted mcDNA polyplexes achieved up to 200-fold higher gene transfer in c-Met-positive prostate cancer cells, highlighting the delivery benefit of mcDNA miniaturization.	No single platform is optimal. Electroporation is efficient but less scalable and poorly APC-selective, while lipid and polymeric carriers offer more flexibility and scalability but need further optimization for toxicity, biodistribution, APC uptake, endosomal escape, nuclear entry, and manufacturing reproducibility [11,92,98,108,109].

**Table 3:** Summary of advantages and limitations of using a minicircle as a DNA vaccine.

Advantages	Limitations
Enhanced safety profile due to the absence of antibiotic resistance genes and a bacterial backbone	Lower production yield compared with conventional plasmid DNA
Reduced risk of insertional mutagenesis (episomal persistence)	Complex and multi-step manufacturing process
Improved transcriptional stability and prolonged antigen expression	Higher production cost under GMP conditions
Stronger humoral and cellular immune responses across infectious and cancer models	Poor in vivo delivery efficiency without specialized carriers
Lower effective dose required for immunogenicity	Dependence on electroporation or nanoparticle-based delivery systems
Reduced CpG-mediated excessive innate immune activation	Regulatory uncertainty due to the limited clinical guide

## Data Availability

No new data were created or analyzed in this study.
